# Reflections on interdisciplinary research in practice: Epistemological conflicts

**DOI:** 10.1007/s13194-025-00685-x

**Published:** 2025-11-07

**Authors:** Merel Talbi, Roosmarijn van Woerden

**Affiliations:** 1 Faculty of Social Sciences and Humanities, Reasoning and Argumentation, CLUE+, VU Amsterdam, Amsterdam, The Netherlands; 2 Amsterdam Institute for Social Science Research, University of Amsterdam, Amsterdam, The Netherlands

**Keywords:** Interdisciplinarity, Epistemic pluralism, Team science, Integration, Epistemic goods, Epistemic friction

## Abstract

Oftentimes, interdisciplinary research is heralded as an effective way to approach complex problems from a diverse range of disciplinary perspectives. However, many scholars of interdisciplinary research agree that doing interdisciplinary work is difficult and prone to failure. In this paper, we argue that this difficulty is better understood in light of a tension between the aim of interdisciplinary integration on the one hand, and the goal of normative epistemic pluralism on the other. This tension, which we believe takes place at the above-disciplines level of interdisciplinary research teams and projects, leads to difficulties in interdisciplinary research in practice. Additionally, we argue that the conflicts on the local, practical level where disciplinary researchers work together in multidisciplinary research teams - which we term the between-disciplines level - are caused by researchers insufficiently acknowledging the differences in (non-)fundamental epistemic goods that vary per academic discipline. In teams that do interdisciplinary research, it can occur that disciplines are brought together that have very different underlying epistemic presuppositions (or epistemic systems) that value different forms of research, methodologies and aims. We illustrate the difficulties of interdisciplinary research with the case study of the Mill Town Example, an interdisciplinary project that was deemed a failure by its participants, in order to sketch what epistemological conflicts look like in practice on the between-disciplines level. In order to address differences in how disciplinary researchers value (non-)fundamental epistemic goods, we suggest various forms of epistemic work that offer strategies to make epistemological conflicts manageable in interdisciplinary research.

## Introduction

Interdisciplinary research is gaining momentum in academia in the last decades, and is often presented as a way to investigate complex problems (Imbruce & Prazak, 2021; Boradkar, 2010). Sometimes, an interdisciplinary approach is even portrayed as being necessary for “solving society’s most pressing problems” (Lattuca et al., 2017, p. 72). The National Research Council of the United States predicts that research will increasingly be interdisciplinary, often conducted in teams of researchers (National Research Council, 2015).

Interdisciplinary research is mainly used to address complex problems that require insights from more than one discipline (Repko & Szostak, 2021), thus presuming a form of epistemic *pluralism*. For interdisciplinarity to truly include various academic perspectives, epistemic diversity is a requirement, where conceptual, material, theoretical, methodological, socio-cultural or institutional differences “affect the development and/or understanding of knowledge” (Leonelli, 2022, p. 997). Pluralism is generally *descriptively present* in interdisciplinary research, as different disciplines are always involved. However, it is also *normatively valued* in interdisciplinary research, because the combination of knowledge and insights from different scientific disciplines and traditions is seen as key to solving complex problems (Chang, 2020; Miller et al., 2008). However, these differences in disciplinary perspectives and disciplinary epistemic systems^1^ create challenges for interdisciplinary research as researchers are firmly rooted in their own ‘disciplinary domain’ and cannot easily understand each other’s epistemic system or work with it (MacLeod, 2018). Such differences in the epistemic systems underlying academic disciplines are enforced in the current discipline-based structures of universities (Bellotti & D’Agostino, 2021, p. 72). Discipline-based thinking, based on disciplinary epistemic systems can and has led to situations where researchers in interdisciplinary projects experience resistance ‘from within’ the project itself, because they face differences and disagreements that are hard to overcome (Imbruce & Prazak, 2021). Interdisciplinary research projects have been characterized by Leahey et al. (2017) as ‘high-risk, high-reward’, because disciplinary diversity may lead to better results when insights are integrated into new knowledge, but it may also lead to misunderstanding, tension, and conflict due to differences in epistemic systems.

The hypothesis that these differences in epistemic systems may lead to difficulties in interdisciplinary research projects, is supported by recent academic literature. Fam and O’Rourke (2021), for example, state that interdisciplinary research and collaboration are often quite complex, making it prone to failure. Other interdisciplinary researchers report that collaboration between social scientists and natural scientists can be especially difficult because of differences in epistemic assumptions and biases (Callard et al., 2015; Fletcher & Lyall, 2021; Imbruce & Prazak, 2021). The difficulty to find commonality becomes greater when epistemic systems of disciplines are further apart, which is referred to as ‘wide interdisciplinarity’ (Repko & Szostak, 2021). As Miller et al. (2008), Callard and Fitzgerald (2015), Mäki et al. (2018) and Healy (2003) note, often this tension over differences ends with one disciplinary perspective becoming dominant, or becoming “epistemically imperialistic” (Miller et al., 2008, p. 46).

In contrast with the difficult practice of interdisciplinary research, in which differences in epistemic systems often lead to disagreements and misunderstandings, the *integration of insights* is often the explicit aim of interdisciplinary research projects. Many scholars (also known as ‘integrationists’) conceptualize integration as the defining characteristic of interdisciplinarity, setting it apart from multidisciplinarity (Repko & Szostak, 2021), but some others (including so-called ‘generalist’ interdisciplinarians) have criticized that integration between disciplinary insights should be at the core of interdisciplinarity (Boon & Van Baalen, 2019). The aim for integration appears to follow from the goal of bringing together different disciplinary insights to study one phenomenon, while also creating a *tension* with the other aim of interdisciplinarity, which is to normatively value pluralism. As Boon and Van Baalen (2019) point out, the aim of integration seems to build on and presuppose a *unity of science* assumption, which is at tension with normative pluralism, where it is precisely the *different forms* of knowledge which are valuable. In this paper, we wish to clarify how we believe the dual aims of integration and normative pluralism lead to this tension at the level of interdisciplinary research projects, how this tension can be better understood by considering epistemological presuppositions between different academic disciplines when individual researchers work together in teams, and how and whether these epistemological differences can be managed. In so doing, we intend to provide insight into one reason why interdisciplinary research may be experienced as difficult by multidisciplinary teams doing interdisciplinary research, and suggest some forms of *epistemic work* that may help researchers in dealing with these difficulties.

Before we move on, let us clarify a basic assumption in our thinking on interdisciplinarity. We accept interdisciplinary research as an existing scientific practice in academia. We also ascribe to the idea that interdisciplinary research can add something to complex scientific questions that purely disciplinary research designs cannot. We see no point in denying the existence or the legitimacy of interdisciplinary research practices. However, we agree with Boon and Van Baalen (2019) that “too little attention is paid to the cognitive and epistemological difficulties encountered by researchers [doing interdisciplinary research], in particular, regarding the question whether the ability to do interdisciplinary research requires a specific understanding of science and related cognitive skills.” (p. 3). In their conceptual work, Boon and Van Baalen (2019) analyze that what they call the physics paradigm of science may hamper understanding of interdisciplinary research as it focuses on reductionism, representation, and the unity of science. They argue that this physics paradigm does not ‘fit’ interdisciplinary research, because it does not allow for pluralism, as it is aimed at unity of science and one ‘right’ representation of the world. Boon and Van Baalen (2019) thus propose an engineering paradigm of science for interdisciplinary research, that allows for an understanding of diverse disciplinary perspectives and pluralism, as it emphasizes how researchers *construct* knowledge (instead of representing an pre-existing, independent reality). We agree with Boon and Van Baalen (2019) that difficulties in interdisciplinary research work become easier to understand when looking at how various disciplinary perspectives construct knowledge *differently*. Their engineering paradigm additionally focuses on building epistemic tools for a certain epistemic task and strives for more practical problem-solving, which allows for localized interdisciplinary projects to focus on specific research questions and results relevant to the project.

Where Boon and Van Baalen (2019) have made important conceptual headway on the epistemological side of interdisciplinary research, we argue in this paper that additional conceptual work is needed, because (a) interdisciplinary research paradigms of science are operating at different levels, (b) epistemological differences between disciplinary perspectives can be further explored to understand the barriers to successful interdisciplinary research, and (c) we need more insight into what might be fruitful ways forward when disciplinary epistemic systems conflict.

First of all, interdisciplinary research could be understood to have two levels of epistemological systems: the *above-disciplines* level of interdisciplinary research projects and the *between-disciplines* level. Boon and Van Baalen (2019) focus on what we call the *above-disciplines* level of interdisciplinary research projects, where they suggest that researchers adopt an overarching epistemological paradigm for a research project before that project commences, and which intends to provide an epistemological framework for the entire project. We argue that paradigms of science also operate on a *between-disciplines* level, where within one interdisciplinary research project different disciplines which may have very different epistemic systems based on different paradigms of science work together on a joint research problem. At this level of interdisciplinary research in practice, individual disciplinary researchers may hold different epistemological presuppositions and epistemic systems (for example with regard to the relevant epistemic resources or the proper aims of scientific research), that can clash while research takes place ‘on the ground’. Disciplines may operate within the physics paradigm of science, as Boon and Van Baalen (2019) call it, while others operate from the engineering paradigm of science. Additionally, many disciplines inhabit different paradigms of science, even within the same discipline, as with more quantitatively-oriented versus more qualitatively-oriented variations of individual social science disciplines. Thus, on the *between-disciplines* level, disciplines can have different paradigms of science, as well as other differences between their underlying epistemic systems.

Secondly, at the *between-disciplines* level, different frameworks for analyzing the (core elements of the) disciplinary perspective exist, such as the framework of Repko and Szostak (2021) and the framework of Van Baalen and Boon (2024). Repko and Szostak (2021) analyze disciplinary perspectives in terms of studied phenomena, underlying assumptions and epistemology, and theories, methods and concepts used in the discipline. Van Baalen and Boon (2024) add goals, objectives, models, practical constraints and pragmatic criteria of the disciplinary perspective. We argue that especially the epistemological aspect – particularly with regard to fundamental and non-fundamental epistemic goods – of these disciplinary perspectives requires more attention.

Thirdly, we argue that some sort of *epistemic work* needs to be done before insights can be combined from the different disciplines that are produced by the different disciplinary epistemic systems. This type of epistemic work is conceptualized by Boon and Van Baalen (2019) in the form of three metaphors of interdisciplinarity, of which they find the engineering design metaphor, which focuses on the construction of epistemic resources for specific epistemic tasks, the most useful. (Boon and Van Baalen define epistemic resources as the epistemic products that disciplines produce, such as scientific knowledge, but also the resources that are used in the production of this knowledge, such as theories, data and models (Boon & Van Baalen, 2019).) We understand this engineering metaphor to include the construction of epistemic resources for epistemic tasks to require *epistemic work*, as disciplinary epistemic systems differ. In this paper, we will provide an account of what this epistemic work might entail (formulated by using concepts from (social) epistemology, such as in the concept of *epistemic goods*, or the ideas of what proper aims for scientific research are (Pritchard, 2014, 2016), and how this epistemic work might in practice be undertaken. Even if integration is not the final aim, working together across epistemic systems requires some sort of agreement on the epistemic presuppositions that are acceptable for all researchers, and this agreement requires *epistemic work*. In addition to clarifying the tension between integration and epistemic pluralism, therefore, this paper also intends to provide an analysis of how the construction of an epistemic system for the specific joint interdisciplinary research task can take different forms (presented in Sect. 4), based on the *type of differences* between the epistemic systems.

To specify these types of differences between disciplinary epistemic systems, we use the concepts of fundamental and non-fundamental epistemic goods (Coliva & Pederson, 2017). Fundamental epistemic goods are also called non-derivative epistemic goods – these are epistemic goods that do not derive their value from another epistemic good but have intrinsic value in and of themselves (Coliva & Pederson, 2017). In contrast, non-fundamental epistemic goods, or derivative epistemic goods, do derive value from a different epistemic good, or its relation to that epistemic good (Coliva & Pederson, 2017). Between disciplines, differences can be characterized as differences over (a) what should be considered a fundamental epistemic good (e.g. truth), (b) the exact nature of a certain fundamental epistemic good (i.e. the nature of truth as ‘representation of reality’), (c) what should be considered a non-fundamental epistemic good (i.e. ‘rationality’), (d) the exact nature of a certain non-fundamental epistemic good (i.e. the nature of rationality as ‘logically valid’).

In this paper, we argue that for every type of difference between disciplinary epistemic systems, a different *type of epistemic work* can be done. For differences over fundamental epistemic goods, the aim of integration seems out of reach, as such fundamental epistemic differences cannot be overcome. In that case, a different project design in which exchange, exploring disciplinary differences and ‘problem-feeding’ are central may be more feasible (Thorén & Persson, 2013; Boon & Van Baalen, 2019). For differences over non-fundamental epistemic goods, in-depth conceptual discussions about these differences may lead to a joint epistemic system for the interdisciplinary research project in which integration is possible. With this analysis of differences in epistemic systems at the *between-disciplines* level, we hope to add to initial conceptual work done by Boon and Van Baalen (2019) on the *above-disciplines* level.

This paper proceeds as follows. In Section 2, we want to clarify the difficulties of interdisciplinary research through concepts from social epistemology, specifically by introducing the important role of fundamental and non-fundamental epistemic goods. In Section 3, we present a real-life case of interdisciplinary teaching, the Mill Town Example, to illustrate how epistemic goods play a role in explaining the tension between integration and epistemic pluralism. In Section 4, we suggest various types of *epistemic work* that can be done to deal with differences between epistemic systems. We end with a brief conclusion.

## Integration, epistemic pluralism and potential epistemic tensions in interdisciplinary research

So far, we have posited that we believe that difficulties in interdisciplinary research are likely caused by a *tension* at the above-disciplines level, that is caused by the dual aims of interdisciplinary integration and epistemic pluralism. The intention of this paper is to clarify this tension, and in addition to this, to better understand the difficulties of interdisciplinary research at the between-disciplines level, where different epistemic systems may clash. In this section, we clarify some of the concepts that we use to analyse this alleged tension in interdisciplinary research, including the concepts of above-discipline and between-discipline levels in interdisciplinary research, integration, epistemic pluralism, and the difference between fundamental and non-fundamental epistemic goods. In addressing these terms, we also clarify how our paper builds on other important pioneering and conceptual work on interdisciplinary research, such as the work of Repko and Szostak (2021), MacLeod (2018) and Boon and Van Baalen (2019).

### Interdisciplinary integration at the above-disciplines and between-disciplines levels

An interdisciplinary research project deals with two levels of epistemic systems, which we term the *above-disciplines* level and the *between-disciplines* level. Firstly, at the *above-disciplines* level, an epistemic system for interdisciplinary research in general is used. Boon and Van Baalen (2019) have rightly pointed out that this *above-disciplines* level epistemic system should allow for plurality, as one of the prime characteristics of interdisciplinarity is that it values the plurality of insights coming from different disciplines. The ideal of normative pluralism requires all relevant disciplines to be included, with *interactive* normative pluralism explicitly valuing that different forms of knowledge and insights from various academic disciplines do *more* than merely exist within a project side-by-side (Chang, 2012). This interactive variant of epistemic pluralism is what we liken to *integration* (which we will discuss below) in interdisciplinary research, where various insights are combined to resolve complex problems by providing new knowledge when insights are combined (Chang, 2012). However, if interactive normative pluralism (since it is similar to integration) is a prime value of interdisciplinary research, then *all* relevant disciplines should be incorporated to reap the benefits of interaction, even if underlying disciplinary epistemic systems may conflict.

At the same time, many in the field of interdisciplinary research hold on to integration as the prime characteristic of interdisciplinarity. Pioneers such as Klein (1990), Bill Newell (2001, 2007), and Allen Repko and Rick Szostak (2021) have argued that one of the main characteristics of interdisciplinary research is the aim to *integrate* knowledge from different scientific disciplines (Klein, 2010; Repko & Szostak, 2021). Repko and Szostak (2021) define integration as “the cognitive process of critically evaluating disciplinary insights and creating common ground among them to construct a more comprehensive understanding” (p. 23). Klein states that interdisciplinarity’s underlying ideas of unity and synthesis require a “common epistemology of convergence” (Klein, 1990, p. 11). The integrationist interdisciplinarians state that in order to create a more comprehensive understanding of a phenomenon, different disciplinary insights cannot be just placed side-by-side, but have to be integrated (Repko & Szostak, 2021), and that this requires an *above-disciplines level* epistemic system that allows for convergence. In addition, integration should take place without preferencing one discipline over another, so the aim of integration is an aim for *balanced* integration, excluding epistemic imperialism where one discipline’s preferred epistemological and methodological views persevere without substantive reason and agreement from the entire team (Miller et al., 2008). This situation of aiming for integration while also valuing epistemic pluralism and wishing to include *all*, including potentially conflicting, academic disciplines, creates a tension at the *above-disciplines* level. Similarly worrying about epistemic imperialism and the difficulty of securing balanced pluralism, Boon and Van Baalen (2019) have criticized the aim of integration with its unity-of-science-like aspirations, and instead opt for the *engineering* paradigm of science. However, the aim of integration is still very central to much scholarly work (including that of aforementioned pioneers like Klein, Newell, and Repko and Szostak) on interdisciplinarity, and so this tension remains for many in the field.

At the *between-disciplines* level, where different researchers work together within a project to contribute their own disciplinary expertise in an interdisciplinary set-up, disciplines operate on the basis of different epistemic systems. The tension at this local level is not between normative pluralism and the aim of integration, but between the disciplinary epistemic systems themselves. For an interdisciplinary project that values epistemic pluralism, disciplines are likely selected based purely on their relevance to the research question. If disciplines are selected based on their relevance to the research topic, disciplines should not be excluded because they do not ‘fit’ with the epistemic systems of other disciplines, and so it is entirely possible that they are selected from different ‘ends’ of an epistemic spectrum. Additionally, if epistemic pluralism is valued, the interaction between disciplinary epistemic systems should be on an equal basis, because if one epistemic system is adopted at the cost of another, the danger of epistemic imperialism arises, where one set of epistemic presuppositions becomes dominant (Miller et al., 2008; Callard and Fitzgerald; Mäki et al., 2018; Healy, 2003; Repko & Szostak, 2021), and relevant insights from other disciplines may be overlooked and may not be incorporated.

Thus, researchers within one interdisciplinary project may draw on knowledge and insights from very different domains, using different theories, methods, and concepts, basing themselves on different epistemic assumptions, and potentially producing conflicting insights (Andersen, 2016; Repko & Szostak, 2021). In other words, researchers are all grounded in their own specific *domains* (MacLeod, 2018) and the epistemic systems underlying these disciplinary domains may be fundamentally in conflict with each other (Miller et al., 2008). On MacLeod’s account, each discipline or domain has a high level of specialization that is aimed only at answering the questions that belong to that discipline, basing research on the epistemic system of that discipline. This specialization works best in the context of research in that discipline, leading to misunderstandings when scholars from one domain try to work with scholars from another (MacLeod, 2018). These misunderstandings often reflect fundamental domain specific differences, such as different ideas about what good science is. MacLeod (2018) concludes that there are no easy fixes to resolve situations of strong clashing domain specificity. A prime example of such a strong clashing domain specificity is when ideas about the nature of ‘truth’ differ fundamentally, which we understand as a situation where fundamental epistemic goods do not align (something that we will turn to in the next section).

In short, this paper aims to understand the difficulties of interdisciplinary research from an analysis of the different types of differences between epistemic systems at the *between-disciplines* level, and offers ways to do epistemic work to deal with those differences, by using the concepts of fundamental and non-fundamental epistemic goods, which we turn to in the next section. From this analysis on the *between-disciplines* level, we also offer some ways to think about the tension between normative pluralism and the aim of integration at the *above-disciplines* level.

### Differences in epistemic systems: fundamental and non-fundamental epistemic goods

The distinction between *fundamental* and *non-fundamental* epistemic goods may be useful to understand what type of differences exist between disciplinary epistemic systems. Traditionally in epistemology, the topic of epistemic goods or epistemic goodness deals with the question of what is epistemically desirable, and what the goal of epistemic inquiry is or should be (Pritchard, 2014, 2016). A variety of different epistemic goods may be important, such as (and often primarily) truth, but also knowledge, justification, reliability, explanatory power, or understanding (Pritchard, 2016). Various accounts then exist to further specify how one can understand these epistemic goods substantively, and, importantly, how various kinds of epistemic goods relate to each other. In evaluating various epistemic goods, one discussion in the literature centres around the question of which epistemic good should be understood to be *fundamental*. *Fundamental* epistemic goods do not derive their value from another epistemic good but have intrinsic value in and of themselves (Coliva & Pederson, 2017), in contrast to *non-fundamental* epistemic goods that derive their value from other goods. Various different types of epistemic goods can be valued by various (sub-)disciplines, such as truth (Pritchard, 2014), knowledge (Goldman, 2011) or understanding (Elgin, 2017). Pritchard (2014), for example, is a great proponent of the view that truth is the *fundamental* epistemic good, or the one epistemic goal that is most important and that other epistemic goods rely upon for their value. Other, non-truth epistemic goods are then relevant mostly in an instrumental manner, or to the degree that their acquisition may lead to a better grasping of the truth (Pritchard, 2014, 2016).

Given the range of various epistemic goods, we believe that it is likely that in interdisciplinary projects researchers may hold different (*fundamental* and *non-fundamental*) epistemic goods.^2^ This may occur when researchers do not share a focus on truth, or have a different conception of what truth is or how it is uncovered. Concretely, this may take the form of a physicist focusing more on truth (some form of correspondence of hypotheses with a realistically-understood empirical world, say), while an anthropologist may strive for a more holistic, integrated form understanding that includes the interpretations and experiences of interviewees. In these situations, differences in research goals, methodologies, theories, concepts, and design will likely follow.

We argue that if differences exist in *fundamental* epistemic goods, the chance of agreeing on a joint epistemic system for the project and balanced integration is very limited (if not non-existent), since these differences are deeply rooted in the epistemic system underlying the discipline. They are, we might say, so grounded in the way the discipline understands the world, that it makes them hard to change, and that they are generally non-negotiable.

Two types of differences in *fundamental* epistemic goods may exist between disciplinary epistemic systems, both of which may prove difficult to overcome. Firstly, there may be a difference in whether an epistemic good (for example, truth) is considered fundamental or not. This type of difference poses a problem for the creation of a joint epistemic system for the research project, as the very aim of the epistemic domain is itself subject to difference and disagreement. Here, we might wonder whether the possibility of a joint epistemic system for the research project may be almost non-existent, unless a form of epistemic imperialism takes place and one discipline simply ‘determines’ that their understanding of this epistemic good must be dominant. While this move would in some ways ‘resolve’ the disagreement, it would not really be in the spirit of interdisciplinary research, which values balanced plurality.

The second type of difference is when disciplines hold the same epistemic good (e.g., truth) to be fundamental, but differ severely in their views on the *nature* of that epistemic good. For example, monists and pluralists hold very different views on the nature of truth, and it seems incoherent to hold that there are several ways of being and knowing X (pluralist) and that there is only one way of being and knowing X (monist) at the same time. This type of difference is less severe than the first type, as there is at least agreement on what the fundamental epistemic good is. However, the example of truth monists and truth pluralists shows that these difference can also run deep, and that the nature of a *fundamental* epistemic good may also prove an obstacle that cannot be overcome to reach the formulation of a suitable joint epistemic system for the research project. Disagreement over fundamental epistemic goods may be expected to occur more often in interdisciplinary research with ‘wide’ interdisciplinarity, which means the epistemic systems of the participating disciplines are epistemologically further apart and do not closely resemble each other (Repko & Szostak, 2021).

Differences in *non-fundamental* epistemic goods may also exist with regard to what can be considered a *non-fundamental* epistemic good, and the *nature* of certain kinds of *non-fundamental* epistemic goods. These differences may be overcome by in-depth discussions, in cases where there *is* agreement over fundamental epistemic goods. In these situations, important presuppositions are already shared, and smaller questions may remain that are more easily resolved (for example, specific methodological choices that do share certain fundamental epistemic assumptions particular to the disciplinary domains in the question). Here the construction of a joint epistemic system and epistemic resources for a specific interdisciplinary research task, as Boon and Van Baalen (2019) envision, may be possible. Using this joint epistemic system may even result in integration of disciplinary insights, although that is not necessarily achieved. These are variants of what we call *epistemic work* that may help to manage the difficulties of interdisciplinary research, and which we will develop further in Section 4.

In short, we believe that the distinction between *fundamental* and *non-fundamental* epistemic goods helps to understand the types of differences between disciplinary epistemic systems, and what types of epistemic work needs to be done to move forward in an interdisciplinary project. We will now illustrate what kind of differences may exist between disciplinary epistemic systems by analysing the Mill Town Example.

## The Mill Town Example: An illustration of a failed interdisciplinary project

In the tradition of the philosophy of science in practice, previous work has been done to study how interdisciplinary research projects actually work, with authors such as Nancy Nersessian, Miles MacLeod, Erika Mattila, Renate Klaassen and Antoine van den Beemt and colleagues undertaking work to map and analyse how various forms of interdisciplinary research function in practice (Nersessian, 2008, 2006; MacLeod & Nersessian, 2013; Klaassen, 2018; Mattila, 2005; Van den Beemt et al., 2020). However, these studies generally focus less on interdisciplinary *failures* and the conflicts that may be the cause of these failures (an exception is the work by Van den Beemt and colleagues (2020), which briefly touches upon institutional and epistemological conflicts in interdisciplinary research). Our paper aims to address these questions of the epistemic difficulties of interdisciplinary research more explicitly. For this reason, we use a case in this paper to illustrate our discussion of epistemic systems for interdisciplinary research projects, the role of normative pluralism and fundamental and non-fundamental epistemic goods. In discussing these topics in the context of this case study, we contribute to both theoretical and practical epistemic questions in interdisciplinary research.

### The Mill Town Example

The case we present here is a collaboration of researchers and educators from different disciplinary backgrounds who designed an interdisciplinary course sequence for a small liberal arts institution in the United States (Imbruce & Prazak, 2021).^3^ The goal was to design a course sequence that was place-based and in which students would learn how to apply academic models and methodologies to the places they live. In this project, Imbruce and Prazak were themselves involved in the design and enactment of the course – which they describe as an ‘interdisciplinary failure’ (Imbruce & Prazak, 2021). The goal was to have students gain a deeper understanding of both particular places, as well as of the concept of ‘place’ in general. The course organisers chose the topic of ‘The Future of the New England Mill Town’, and students were asked to explore what development opportunities could be identified for these towns. The project team wanted to bring together an “understanding of the natural environment, human values and desires and historical and contemporary issues of importance to the locale” (Imbruce & Prazak, 2021, p. 181) and therefore brought together faculty from different disciplines for the course development and implementation. At a broad level, the project team was also interested in merging STEM, social sciences and humanities approaches, bringing together a focus on empirical data analysis with an understanding of meaning and context. The underlying idea for this combination of approaches was that STEM’s empirical process based on causal analysis and identifying general patterns is very different from interpreting how meaning and values are formed in human experience. This interdisciplinary combination of perspectives suggests that the course developers believe that both kinds of understandings are needed to tackle complex problems. In order to provide this variety of perspectives, the course’s team consisted of a chemist, a social psychologist, a geologist, an architect, an ecologist, an anthropologist and an economic botanist. In the process of the course, the collaborators had in-depth discussions over basic definitional issues and more fundamental questions on how to understand the concept of ‘place’.^4^

In their description of this interdisciplinary endeavour, Imbruce and Prazak (2021) present a variety of problems that occurred in the teaching of the course. First of all, they encountered fundamental differences in perceptions of *validity of different types of knowledge* between faculty from the STEM sciences and the social sciences and humanities (SSH), which led to a failure to combine and integrate different approaches. The authors state that it is a challenge in interdisciplinary collaboration to look beyond one’s own disciplinary stronghold and to identify where commensurability or complementarity can be found. They found it difficult to counteract the tendencies towards disciplinarity and the separation of modes of knowing within academia, even though their very aim was to synthesize disciplinary perspectives. Over the course of the project, the authors claim they never quite succeeded to do so, instead being mired in fundamental epistemological discussions:

We found the debate over the validity of knowledge – between disciplines and sectors – to be the primary point of negotiation, rather than the content and pedagogical approaches of the course sequences, the topics that the course development process was meant to discuss. (Imbruce & Prazak, 2021, p. 190)

In hindsight, the authors explain that they were not prepared for the disparities in their ways of thinking, or for the hidden rigidity of structures that undermined their group project: “The hierarchy of knowledges operating within our group, with science seen as more reliable and human-centred approaches as more ‘squishy’, led to […] failure” (Imbruce & Prazak, 2021, p. 193). Fundamental tensions between the STEM disciplines and the SSH could not be resolved, and the group failed to overcome disciplinary hierarchies that favoured the knowledge and methods produced by the STEM disciplines as the most valued. The natural sciences gained primacy within the group, which made co-production of knowledge very difficult or even impossible. Often, the suggestions by the SSH team members were deemed ‘not scientific’ by colleagues from the STEM disciplines:

[The] understanding [of the colleagues from the Science faculty] of the common goal was to make everyone think like a scientist and to call into question any proposal or understanding which according to them was not “scientific.” As a result, we often heard the refrain, “that’s well and good but what does that have to do with science?,” and in many cases there were complaints that the questions, readings, and activities proposed or undertaken were not “scientific.” (Imbruce & Prazak, 2021, p. 184).

Secondly, Imbruce and Prazak (2021) did not predict that in an interdisciplinary project, disciplinary needs or convictions would be considered more important than interdisciplinary initiatives. During the project, it became clear that some of the colleagues did value an interdisciplinary or multidisciplinary approach, while some others did not (or, at least, less so). This created a ‘fault line’ where the STEM scientists relied on quantitative research methods and (in the analysis of Imbruce and Prazak) valued interdisciplinarity less than the colleagues from SSH, who used qualitative research methods. This caused the groups to drift apart, leading to a divide that was hard to overcome: “This fault line caused subgroup formation and there was reduced trust and communication between the two subgroups, the quantitators and the qualitators.” (Imbruce & Prazak, 2021, p. 184).

Finally, when the authors realized there was an intellectual divide which created serious obstacles to the collaborative process, they were unable to formulate and enact strategies to overcome this divide, finally leading to a suboptimal result. Imbruce and Prazak (2021) concluded that in practice, it is very difficult to truly integrate knowledge from varying intellectual traditions. For future projects, they conclude that for interdisciplinary endeavours to work, it is important to have a *broader* sense of what a successful interdisciplinary project is: “To fully evaluate an interdisciplinary project, communicative, value-driven and process-oriented goals need to be articulated, and not just goals that can be assessed through quantification.” (p. 194) Ideally, the definition of the success of an interdisciplinary collaboration should be more than just the output or product.

### What went wrong in the Mill Town Example?

If we are to believe Imbruce and Prazak, the Mill Town educational project was an interdisciplinary failure. How can this failure be accounted for? Based on the theoretical conceptualizations we have suggested in the previous section, we argue that, among other reasons, various disciplinary differences and disagreements led to the Mill Town project not living up to the expectations of the participants.

First, it appeared that there were insufficient explicit attempts to construct epistemic resources for a specific epistemic task (Boon & Van Baalen, 2019) and a joint epistemic system for the project. The disciplinary differences were not fully explored, and it was not clear to the team with what type of differences between epistemic systems the team was dealing. They also did not appear to have done sufficient *epistemic work* to deal with those differences. While Imbruce and Prazak do describe that definitional discussions took place amongst the team members (including on the concept of ‘place’, which was very important for the project), there did not appear to be any kind of collaborative construction on a joint epistemic system for the project, nor on epistemic resources they could jointly use. In fact, from Imbruce and Prazak’s description of, it almost appears like the Mill Town course was more *multidisciplinary* in its set-up. This is visible in the way the SSH and STEM scholars relate to each other, especially with regard to how the researchers value the various research methods that they use. The disdain that appears to speak from some of the interactions between the team members from different disciplines shows some of the *domain specificity* that MacLeod (2018) describes in his work, with strongly different ideas about what good research is, what valuable knowledge is, and what legitimate research methods are. From the framework of Boon and Van Baalen (2019), it can also be said that at the *between-disciplines* level, both the engineering as well as the physics paradigm of science were represented in the disciplinary epistemic systems present, which led to disagreements between representatives (researchers) of the different epistemic systems. Although, again, conversations were had on these topics, the descriptions of Imbruce and Prazak do not suggest that there was any real wish on behalf of the team members to really do *epistemic work* in order to deal with any of these differences.

One way to understand why these discussions did not resolve the existing disagreements is by acknowledging that the differences between the epistemic systems of the various researchers were too large to bridge, and that the Mill Town project was an instance of *wide interdisciplinarity*. In this variation of interdisciplinary research, the differences between the participating academic disciplines and their underlying epistemic systems were perhaps simply too great, which led to a situation where dialogues to create common ground between disciplines and researchers failed. The differences between epistemic systems was too great at the *between-disciplines* level, but this also had implications for the *above-disciplines* level, as the team resolved these differences by epistemic imperialism, thus violating the normative pluralism as well as the aim of balanced integration of the *above-disciplines* level epistemic system of interdisciplinarity.

This apparent lack of wanting to resolve the existing tensions is explained by Imbruce and Prazak by pointing to some team members not really *valuing* the interdisciplinary approach of the Mill Town project. This might be understood in an institutional vein, and we may wonder whether team members were appropriately rewarded for their interdisciplinary efforts by their own research and teaching institutes, or whether this interdisciplinary project is merely seen as a frivolous caper that does not truly produce any interesting results. Fundamentally, however, even this kind of institutional explanation may hint at a lack of *normative epistemic pluralism* that underlies the Mill Town project, with various scholars not truly believing that an interdisciplinary approach leads to knowledge that can address the complexity of certain phenomena.

If we want to look towards strategies to analyse the tensions that surfaced in the Mill Town Example, an analysis of the fundamental epistemic goods for the various disciplinary scholars (or their domains, as MacLeod would say) might prove a good starting point. Again, these epistemic goods describe what various researchers understand the goal of their research enquiry to be. Potential epistemic goods may be knowledge, understanding, reliability, justification, explanatory power, or truth – although this list is not exhaustive, and differences may exist in how these concepts are then cashed-out across various academic disciplines and their underlying epistemic systems. In the Mill Town Example, the authors describe that they discovered fundamental differences between collaborators from different disciplines on which form of *knowledge* is more *valid*. They describe how a hierarchy of types of knowledge was formed in their group, in which knowledge production in the STEM sciences was seen as *reliable* and human-centered approaches as more *‘squishy’*.

In these descriptions, it seems that the epistemic goods between the various researchers differed considerably, with STEM and SSH team members holding varied ideas about what kinds of knowledge were a desirable end product for the project. Variations come to the surface with regard to reliability and validity – two examples of epistemic goods that are mentioned by Pritchard (2014) in his work on epistemic goods, and which are also mentioned explicitly by Imbruce and Prazak in their analysis of why they believe the Mill Town project failed. Imbruce and Prazak also mention that there were negotiations between the researchers on how to understand the validity of the knowledge that was being produced, and that this was even the primary point of contention within the project. The centrality of this discussion on exactly which epistemic goods should be sought in this interdisciplinary project, and which type of *knowledge* would be *valid* and why (again, two examples of epistemic goods), all point towards different ideas on which epistemic goods should be deemed *fundamental* within the project. The fact that there is disagreement on which epistemic goods should be aimed at above all others, appears to point in the direction of not only a disagreement on which epistemic goods are important, but even on a very fundamental disagreement on epistemic goods – a disagreement for which the Mill Town team did not appear to have any constructive approaches for resolving. This lack of effective strategies to manage the differences may have stemmed from an inability on the researchers’ parts to formulate their disagreement in terms of fundamental epistemic goods or differing epistemic systems, and an inability to adapt the kind of *epistemic work* they were doing to the type of difference in epistemic systems they were facing. They resorted to a solution that does not require *epistemic work*: domination of one epistemic system over the other.

Interestingly, Imbruce and Prazak conclude their analysis by claiming that the interdisciplinary Mill Town project failed also due to an insufficiently *broad* understanding of the aims of the project. Again, this hints towards a project where there was insufficient agreement about epistemic goods, and the absence of a joint epistemic system where these varied epistemic goods are discussed and (hopefully, in the end) agreed upon. Imbruce and Prazak even go so far as to say that these broader aims should include “communicative, value-driven and process-oriented goals”, which go far beyond the more ‘technical’ epistemic goods that Pritchard mentions as the traditional goods that epistemologists are generally interested in. These epistemic goods that focus more on cooperation, communication and values show that successful interdisciplinary research requires more than mere discussions about the particular assumptions, methods, theories and insights of the epistemic systems underlying academic disciplines, but that a broader, more contextual approach is required that also addresses non-epistemic aspects. This aligns with the three types of *epistemic work* that we will develop in the next section, and which presuppose that while differences in epistemic systems may exist, that institutional, social, communicative and value-driven approaches are required to fruitfully discuss and manage the tensions that rise when epistemic systems clash in interdisciplinary projects. This also shows that while we argue that a better understanding of clashing epistemic goods may help to further understand why interdisciplinary projects fail, we do not believe that a sole focus on these epistemic goods can explain these failures in their entirety.

Of course, this all remains an analysis ‘from the outside looking in’, and it is difficult to know what exactly went on within the Mill Town project. Regardless, it seems likely that this project would have benefitted from explicit and structured discussions on epistemic goods, presuppositions and expectations from the beginning, to at least bring any implicit differences in epistemic systems out into the light. These discussions may have been a starting point to think about what kind of *epistemic work* could be done and what achievable interdisciplinary aims might have been for the project – something that appears absent in the description of the example by Imbruce and Prazak (2021). We will now turn to the different types of *epistemic work* that could be done, in light of different types of differences between epistemic systems.

## Differences in epistemic systems and types of epistemic work

In this section, we argue that there are three types of *epistemic work* that can be done to resolve differences between epistemic systems, depending on the *type of difference* between epistemic systems. We argue that differences on whether an epistemic good is fundamental or over the exact nature of *fundamental* epistemic goods require different *epistemic work* than differences in *non-fundamental* epistemic goods. In this section, we elaborate upon what form this epistemic work may take, and what effect this may have in interdisciplinary research projects.

### Differences in fundamental epistemic goods

Firstly, if there are differences on whether an epistemic good is fundamental or over the exact nature of *fundamental* epistemic goods, the conflicting epistemic systems cannot be aligned in a joint epistemic system for the project. The fundamental methodological and conceptual domain specific differences MacLeod (2018) identifies may simply prove impossible to overcome, as these fundamental goods are non-negotiable and can therefore not be adjusted into a new joint epistemic system for the project. At a *between-disciplines* level, this means that it will be hard to construct joint epistemic resources for the specific task, as the non-negotiable differences will cause problems at every decision in the project, as can be seen in the Mill Town Example. To overcome this problem, different disciplines can divide research tasks and work more side-by-side, focusing on ‘their’ part of the problem.^5^ By abandoning the construction of a joint epistemic system and joint epistemic resources for the task, each discipline can use its own methods and underlying epistemic assumptions, without the need to resolve non-negotiable fundamental epistemic differences. At the *above-disciplines* level, this will mean that the aim of integration will not be met, as integration relies on a joint epistemic system for the project. What can be upheld at the *above-disciplines* level, is normative pluralism.

The second kind of *epistemic work* that can be done is not aimed at creating a joint epistemic system for the project, but to develop a way to fruitfully exchange between fundamentally different disciplinary epistemic systems, for example by ‘problem-feeding’ each other, which entails the process of researchers learning to recognize new research problems by learning from other disciplines (Thorén & Persson, 2013; Boon & Van Baalen, 2019). In this variation, the differences and disagreement are made explicit and visible, and can themselves become a valuable outcome of the interdisciplinary research process. At first glance, this alternative ‘product’ may appear dissatisfying, as it belies a more modest expectation of the possibilities of interdisciplinary research. However, the emphasis on shared insights and on the value of discussion and deliberation can be compared to how other philosophical fields deal with disagreement. In the tradition of deliberative democracy, for example, and more specifically in the subfield of agonistic democracy, the work of political theorist Chantal Mouffe assumes fundamentally pluralist societies, where the existence of differing values will always lead to conflict (Mouffe, 2000). The way to deal with this conflict is not, she says, to pretend that it does not exist. In fact, attempts to find democratic resolutions for this conflict (as in parliamentary politics) will only work temporarily. Instead, we must find ways to fruitfully discuss our different perspectives, where the crucial point is that our discussion continues, and that we avoid the trap of considering our opponents in debate to be our enemies in arms. Similarly, our goal in interdisciplinary research may not always be to (re)solve the differences between fundamentally different disciplinary epistemic systems, but sometimes rather to *facilitate the situation* where these varying perspectives come into contact with each other (something that, in scientific practice, is still the exception rather than the norm). In fact, wishing to ‘solve’ these issues may only work to illegitimately suppress certain views. We saw this in the Mill Town Example, where researchers from the SSH were considered to produce inferior work that was not considered ‘properly scientific’ by the STEM-colleagues (Imbruce & Prazak, 2021).

Medina (2011), similar to Mouffe, claims that *epistemic friction* is itself a goal (Medina, 2011). Divergent views and discussions can help us to gain a deeper understanding of the important questions in research, while simultaneously preventing tunnel-vision and exclusion of relevant views. With this strategy, visible and explicit disagreement becomes a fundamental part of the process of interdisciplinary research, and the discussions about different fundamental epistemic presuppositions are understood as an important added value of this process (Litsou et al., 2020). Researchers can use epistemic differences and friction to explicitly reflect on (the insights from) their own discipline, and on science in general. In this way, a lot of valuable and legitimate interdisciplinary epistemic work can be done, even if differences between epistemic systems cannot be solved at the between-disciplines level.

### Differences in non-fundamental epistemic goods

Differences between epistemic systems over *non-fundamental* epistemic goods may be less deep than over *fundamental* epistemic goods, as *non-fundamental* epistemic goods are negotiable and can therefore be adapted. This makes room for a jointly constructed epistemic system and epistemic resources for a specific research task (Boon & Van Baalen, 2019), although it is of course no guarantee that such a joint epistemic system is successfully constructed, as that will depend on the quality of the discussion and negotiation. At a *between-disciplines* level, this means that differences in disciplinary epistemic systems can – after explicating disciplinary perspectives (Van Baalen & Boon, 2024) and in-depth discussions over the nature of the differences – can be overcome, when disciplinary epistemic systems are combined into a joint epistemic system for the project. This requires epistemic work in the sense that differences between disciplinary justification, reliability, evidence, reasons, theory, method, concepts, assumptions, values, outcomes and process need to be discussed (Coliva & Pedersen, 2017; Litsou et al., 2020; Repko & Szostak, 2021). For each of these differences, a decision has to be made about what the joint epistemic system will look like and how to adapt and align the disciplinary epistemic systems to make its adoption possible. The envisioned end is not a new epistemic system in which all disciplinary epistemic systems are combined, but rather a *joint* epistemic system that works *for the particular project and the disciplinary scholars included within it*. This approach somewhat resembles Sandra Mitchell’s (2003) call for a piecemeal epistemology, where she claims that conflicts in interdisciplinary research are best dealt with on a very practical level – which might, for example, be centered around a discussion about the preferred products or outcomes of the research project. However, our suggestion focusses more on explicitly formulating an epistemic system for the project, rather than on the relevant part of a practical, methodological research process (although these may of course be intertwined, since practical methodological decisions often do flow from epistemic presuppositions (Mitchell, 2003). For our suggestion, a project’s joint epistemic system will then become the product of a team *negotiation*, in which understanding each other’s epistemic starting point is vital (Eigenbrode et al., 2007). This negotiation is only possible because the differences are non-fundamental and therefore *negotiable*. Of course, as indicated by the work of Fam and O’Rourke (2021), these dialogues, discussions and negotiations may not always be successful, for many different kinds of reasons. In order to provide a more fine-grained insight into the practice of what epistemic work might look like, we will now turn to three different examples of how this *epistemic work* of negotiation can be done.

The idea of interdisciplinary negotiation is present in the literature produced by the Toolbox Dialogue Initiative (TDI), spearheaded by Michael O’Rourke, where emphasis is placed on the epistemic worldviews (defined as the set of commitments with regard to how researchers take the world to be and what is sought to be known about it (O’Rourke & Crowley, 2013) – something closely akin to what we understand epistemic systems to be in this paper. While researchers within disciplines share fundamental assumptions and values concerning the scientific process and do not discuss them much, Eigenbrode et al., (2007) and O’Rourke and Crowley (2013) argue that interdisciplinary research requires explicit discussion of these assumptions and values, as failure to understand them will likely impede an interdisciplinary research process. O’Rourke and Crowley (2013) explicitly position the TDI as an exercise in applied epistemology, where philosophical discussion is undertaken to manage the ‘epistemically multicultural character’ of interdisciplinary research (O’Rourke & Crowley, 2013). They take these challenges to be mainly *communicative* in character (whereas MacLeod considers them to be more fundamentally epistemological), leading to the suggestion that greater mutual understanding about the fundamental epistemic worldviews of different participants in the interdisciplinary project will help the research process. This is achieved through ‘structured dialogue’, where participants are invited to reflect upon their epistemic presuppositions or ‘research philosophies’, with the goal of finding out if interdisciplinary researchers might speak and think differently about what they believe the research problem to be, and how they think it should be studied. For O’Rourke and Crowley, getting to the core of these often hidden philosophical differences is fundamental, because they likely lead to differences in methodological commitments and research standards. While they admit that not *all* challenges will be addressed through these philosophical discussions, they do provide examples of workshops where they have helped to create a shared ‘research language’, where terms that were previously used differently by different disciplinary researchers were re-defined to create a shared lingo (O’Rourke & Crowley, 2013, p. 1945). In our terms, this might be an example of doing the *epistemic work* of creating a joint epistemic system for the project by negotiating non-fundamental epistemic goods.

Another example of this *epistemic work* may be found in feminist literature, in which discussions about differences between epistemic systems is also framed in the light of differences between *value* systems. Taking its cue from feminist philosophers of science (Elliott, 2011), this strand of philosophy addresses how scientists have values that govern their scientific conduct, research design and what they consider ‘good science’. Longino (1987) distinguishes between two types of values that influence science: (1) constitutive values, to establish what constitutes acceptable science, and (2) contextual values, such as personal, social and cultural values. Following Longino’s categorization of values in science, Elliot (2011) concludes that epistemic values (such as explanatory power and predictive accuracy) are generally accepted as legitimate in scientific reasoning, and that even non-epistemic values (political, social or ethical considerations) can influence the ethical considerations of methodology and the selection of research questions (Elliot, 2011). What is still under debate, however, is the extent to which non-epistemic values can play a valid role in traditionally ‘scientific’ parts of the research process, such as in justification (Elliot, 2011). However, if science is understood as inherently value-laden, values may take up a more prominent role in the discussion of scientific practice, and these may even serve as explicit guiding principles, rather than merely implicitly influencing research. If we follow this way of thinking, interdisciplinary integration would again be about *negotiating* a common value system for the research project, and negotiations on these values should be at the forefront of the *epistemic work* that needs to be done to create a joint epistemic system for the project. Of course, value differences on what ‘good science’ is can also be on a *fundamental* instead of *non-fundamental* level, which would mean that a different kind of epistemic work is needed, as discussed above.

Yet another example of *epistemic work* can be derived from the work of Islam (2013). Islam focuses on how storytelling and narratives can be used to negotiate different conceptions of student care in universities, and explicitly describes this process of joint sensemaking as a *negotiation* between different ‘epistemic lenses’ (Islam, 2013). Islam argues that storytelling is especially useful for situations where (empirical) evidence cannot resolve differences, since entire disciplines have systems of evidence that legitimate varied ways of understanding and researching the world (Islam, 2013). These situations are *epistemic impasses*, Islam says, because these differences show us that there are in fact different ways of understanding the world that are all potentially sensible. For Islam, the way out of these impasses is through creative sensemaking, where different elements from diverse ways of interpreting the world (Islam refers to these structures of interpretation as Foucauldian *epistemes*, which we consider to be sufficiently similar to how we use the concept of epistemic systems) can be mixed to create “hybrid solutions to epistemic problems” (Islam, 2013, p. 33). Storytelling is especially useful for this endeavor, because stories are more open-ended due to their being about certain individuals or groups in certain places and times. This means that general rules or universalities that are at play in narratives will always have to be applicable to certain specific *situations*, Islam argues, which make them particularly suited to resolving epistemic tensions in institutional settings (Islam, 2013). Because of this, stories also naturally leave room for different characters, perspectives and beliefs and allow these differences to exist side-by-side, and to make sense *despite* their differences. This pluralism makes it possible to have a sense of coherence while maintaining an open discussion, allowing for a situation where both the various parties who make the story together can hold onto their own self-understanding, while also creating a sense of bonding or community (Islam, 2013, p. 41). While all this may sound rather abstract, Islam illustrates his theory with a case study of a university team addressing questions of student care, and details how the formulating and expressing of stories about the different approaches to student care and the new, envisioned, combined aim that the team was working towards, greatly improved both cooperation and performance. For our context of interdisciplinary research, this storytelling approach may appear unsuited to help with negotiations over fundamental epistemic goods, but it can provide a way into bridging epistemic divides in negotiating over non-fundamental epistemic goods, where differences between disciplinary epistemic systems are smaller, and a *joint story* about a shared epistemic system is required to give different researchers a sense of what role they play in that joint epistemic system, and what that system itself aims to achieve.

On the *above-disciplines* level, these discussions over a joint epistemic system or a joint value system are in line with the normative pluralism of interdisciplinarity, as long as the discussion and negotiation leads to a balanced epistemic system, and disciplinary imperialism has not taken place (Miller et al., 2008; Callard & Fitzgerald, 2015; Mäki et al., 2018; Healy, 2003; Repko & Szostak, 2021). When a joint epistemic system is successfully created for the specific task and joint epistemic resources have been developed, this also paves the way for the aim of integration of insights. If there is a joint epistemic system for the project to build on, this can also provide guidance in how to integrate insights from the different disciplines. When differences *between* disciplinary epistemic systems are over *non-fundamental* epistemic goods, both normative pluralism and the aim of integration can be upheld at the *above-disciplines* level.

## Conclusion

The Mill Town Example shows quite vividly how difficult interdisciplinary collaboration may be, and how hard it is to overcome differences in epistemic systems. We have argued that it is important to acknowledge the types of differences between epistemic systems and to explore these difficulties conceptually, in order to be able to take them seriously and address their root causes.

In this paper, we have addressed what makes interdisciplinary research so difficult on both the *between-disciplines* and the *above-disciplines* level. At the *between-disciplines* level there is a tension between the epistemic systems of the different disciplines, that may differ on what they consider *fundamental* and *non-fundamental* epistemic goods and the nature of these epistemic goods. At the above-disciplines level, there is a tension between the normative pluralism of interdisciplinarity and the aim of integration. By analysing how various types of epistemic differences can be addressed by doing epistemic work at the *between-disciplines* level, we have also shed light on how the tension between normative pluralism and the aim of integration at the *above-disciplinary* level may be addressed. Differences on fundamental epistemic goods will prevent researchers from creating a joint epistemic system and joint epistemic resources for a specific project, because differences run deep and are non-negotiable. Integration will no longer be possible, but normative pluralism can be upheld by using epistemic friction to reflect upon one’s own disciplinary epistemic system. Differences on non-fundamental epistemic goods can be negotiated, and there is a possibility to develop a joint epistemic system and joint epistemic resources for a specific interdisciplinary project. If this joint epistemic system is constructed in a balanced way by including the appropriate kind of epistemic work in interdisciplinary research projects, the risk of disciplinary imperialism decreases, and normative pluralism at the *above-disciplines* level is secured. In so doing, the aims of interdisciplinary research can be safeguarded, despite the many difficulties that these interdisciplinary collaborations might face in practice.

## Data Availability

Not applicable.

## Footnotes

1 Boon and Van Baalen (2019) use the concept of disciplinary perspectives, which they define as a: “more or less coherent set of knowledge, beliefs, values and methods that has become ‘second nature’ in the sense that experts are hardly aware of how the specificities of their disciplinary contribute to the ways in which they do their research and generate epistemic results” (p. 21). We use the concept ‘disciplinary epistemic system’, which is very much in line with their definition of ‘disciplinary perspective’, which we understand to denote the epistemic elements underlying the practices and considerations of a particular discipline. The concept of epistemic systems is developed and used by various authors within social epistemology. Goldman (2011), for example, describes an epistemic system as “a social system that houses a variety of procedures, institutions, and patterns of interpersonal influence that affect the epistemic outcomes of its members” (p. 13), such as science, education, or journalism. Boghossian (2011), in the same volume, defines epistemic systems as “fundamentally different ‘grids’ for determining ‘what sorts of evidence there could be for statements (…)’” (p. 40). In short, both Goldman and Boghossian understand epistemic systems as ways to determine the truth or falsehood of certain practices or statements within a certain social community. We use the term epistemic system similarly, to describe how different academic disciplines develop knowledge that is deemed valuable. These varieties lead to the question whether an epistemic system that unites different disciplinary epistemic systems is feasible, and if it is, what such an interdisciplinary epistemic system might look like. While the concept of epistemic systems may appear to overlap with academic disciplines, we understand epistemic systems to only indicate the *epistemic* elements underlying the practices and considerations of a particular academic discipline. While disciplines may also entail institutional and social elements that to not directly and substantively influence the production of knowledge within a discipline (the social and hierarchical relations between colleagues, say, or the salaries that are paid to researchers), we use the concept of epistemic systems to indicate only those epistemic procedures, institutions, and interpersonal patterns that are directly related to the knowledge that a discipline produces. These epistemic aspects may include things like the phenomena, assumptions, methods, theories, concepts and insights that may lead to conflict in interdisciplinary teams (Repko & Szostak, 2021). When we use the term *interdisciplinary* epistemic system, we refer only to those interdisciplinary collaborations where researchers from various disciplines come together to work on a particular project. We consider fields such as biochemistry, neurobiology or quantum computer science to be *interdisciplines*, which may or may not be interdisciplinary. Many of these interdisciplines function as ‘new’ academic disciplines that make use of a coherent epistemic system where researchers hold a shared view on the relevant phenomena, assumptions, methods, theories, concepts and insights that are central to their ‘new’ interdiscipline (Repko & Szostak, 2021).

2 The concept of epistemic goods is similar to that of epistemic value. Occasionally, the former is used to describe more precisely what the proper *goal* of inquiry should be, while the latter is concerned with how, in different situations, we might value or evaluate the endeavours to reach this goal (Pritchard, 2014). The epistemic value of a process of knowledge production (like interdisciplinary research) may also be weighed against other, non-epistemic values, such as ethical or practical values. We will discuss the role of epistemic and non-epistemic values in interdisciplinary research further in Sect. 4.

3 The Mill Town Example provides a case study that describes an interdisciplinary *educational and teaching* project, and not an interdisciplinary *research* project. Of course, much of what we discuss in this paper relates explicitly to interdisciplinary research projects, and so it may seem that our choice for this case is unusual. However, we believe that many of the fundamental epistemic questions and challenges that we address are equally relevant for interdisciplinary research and interdisciplinary education. It may even be argued that it is *easier* to overcome the tensions between integration and normative pluralism in educational contexts, than it is to do so in research contexts. After all, it may be easier to agree across disciplines that it is useful for students to gain knowledge and understanding of interdisciplinary topics in their education, than it is to agree that interdisciplinary research more adequately addresses complex problems than disciplinary research does. This would make it even more salient that even in an educational context, interdisciplinary cooperation is difficult to achieve. In any case, we believe that interdisciplinary teaching projects are sufficiently similar in the emergence of the relevant epistemic tensions that we discuss here, that this case study can function to illustrate and shed light on interdisciplinary tensions, and, more specifically, on our conceptual analysis of these tensions.

4 This project had transdisciplinary aspects as well, as stakeholders from the mill town were involved in the creation of the course. However, the ‘failures’ described are mainly ascribed to the interdisciplinary process, and as we are primarily interested in interdisciplinary interactions, we have decided to set aside the transdisciplinary aspects, though these may indeed be interesting for philosophical projects that are directed mostly at the epistemic challenges that arise in transdisciplinary endeavours.

5 This may seem a step down from interdisciplinary research, but as Eigenbrode et al. (2007) point out, “a suitable level of integration will depend on the problem and on the mutual understanding of the disciplines involved.” (p. 55). If this mutual understanding is too difficult to achieve, a multidisciplinary approach may prove to be more feasible and apt. As Fletcher and Lyall (2021) observe, the ‘integrative-synthesis’ mode where interdisciplinarity might be conceived in terms of the integration of all the contributing disciplines in a somewhat equal form has proved unrealistic in the context of many collaborations between SSH and STEM. They state that “perhaps we need to accept that, in many cases, a multidisciplinary approach may have to be good enough because otherwise we are being set up to fail.” (Fletcher & Lyall, 2021, p. 54).
